# MLSA phylogeny and antimicrobial susceptibility of clinical *Nocardia* isolates: a multicenter retrospective study in China

**DOI:** 10.1186/s12866-021-02412-x

**Published:** 2021-12-13

**Authors:** Ming Wei, Xinmin Xu, Jingxian Yang, Peng Wang, Yongzhe Liu, Shuai Wang, Chunxia Yang, Li Gu

**Affiliations:** 1 Department of Infectious Diseases and Clinical Microbiology, Beijing Institute of Respiratory Medicine and Beijing Chao-Yang Hospital, Capital Medical University, 8 Gongren Tiyuchang Nanlu, Chaoyang District, Beijing, 100020 People’s Republic of China; 2 Department of Clinical Laboratory, Beijing Ditan Hospital, Capital Medical University, Beijing, People’s Republic of China; 3 Department of Clinical Laboratory, Aerospace Center Hospital, Beijing, People’s Republic of China

**Keywords:** *Nocardia*, MLSA, Antimicrobial susceptibility, Drug susceptibility pattern, China

## Abstract

**Background:**

With the increase of detection rate and long treatment period, nocardiosis has become a noticeable problem in China. However, there are limited large-scale studies on the epidemiology and antimicrobial susceptibility profiles of clinical *Nocardia* spp. in China. The present study aimed to explore the species distribution and drug susceptibility pattern of 82 clinical *Nocardia* isolates from three tertiary hospitals in China by multilocus sequence analysis (MLSA) and broth microdilution (BMD) method.

**Results:**

Pulmonary nocardiosis (90.2%) was the most common clinical presentation of infection. *N. cyriacigeorgica* (*n* = 33; 40.2%) and *N. farcinica* (*n* = 20; 24.4%) were the most frequently encountered *Nocardia* species, followed by *N. otitidiscaviarum* (*n* = 7; 8.5%), *N. abscessus* (*n* = 5; 6.1%), *N. asiatica* (*n* = 4; 4.9%), and *N. wallacei* (*n* = 4; 4.9%). Trimethoprim/sulfamethoxazole (SXT) remained high activity against all *Nocardia* isolates (susceptibility rate: 98.8%). Linezolid and amikacin were also highly active; 100 and 95.1% of all isolates demonstrated susceptibility, respectively. Except for *N. otitidiscaviarum*, all the *Nocardia* isolates exhibited high susceptibility rates to imipenem. The resistance rates of all isolates to clarithromycin and ciprofloxacin were 92.7 and 73.2%, respectively, but the resistance rate of *N. farcinica* to ciprofloxacin was only 25%.

**Conclusions:**

The clinically isolated *Nocardia* spp. had diverse antimicrobial susceptibility patterns, which were similar to the reports by other groups elsewhere, but some differences were also observed, mainly including imipenem and ciprofloxacin. According to this study, SXT still can be the first choice for empirical therapy due to the low resistance rate. Linezolid can be chosen when a patient is allergic to SXT, and amikacin and imipenem can be the choice in a combination regimen.

**Supplementary Information:**

The online version contains supplementary material available at 10.1186/s12866-021-02412-x.

## Background

The genus *Nocardia* which is filamentous gram-positive bacterium and belongs to aerobic actinomycetes, exits in a wide range of environments [1–3]. More than 50 species can cause infections both in immunocompetent and immunocompromised individuals [4]. Due to the inexperience of the clinical laboratory technicians in primary hospitals in China, the particular characteristics of *Nocardia* growth, the unspecific pulmonary symptoms, as well as the low sensitivity of the culture-based method for diagnosis of nocardiosis [5–7], it is easy to miss detection of *Nocardia* in clinical practice. Partially, as a result, *Nocardia* infection is underestimated in China.

*Nocardia* species-level identification plays a crucial role in clinical therapy because particular species have specific drug susceptibility patterns [8, 9], but it is always a complex problem in clinical practice. With the development of matrix-assisted laser desorption ionization-time of flight mass spectrometry (MALDI-TOF MS), the common clinical *Nocardia* species (e.g., *N. cyriacigeorgica* and *N. farcinica*) can be identified rapidly. However, the available database of MALDI-TOF MS is limited for *Nocardia* strains leading to some uncommon isolates with no or false identification [10, 11]. The 16S rRNA gene sequencing is generally considered as the primary means for accurate identification of the clinically encountered *Nocardia* isolates [12], but it cannot discriminate closely related species due to high conservation, unless it combines with a housekeeping gene, such as *gyrB* or *rpoB* [13]. In recent years, multilocus sequence analysis (MLSA) has become more and more critical in bacterial taxonomy and species identification [14–16], because it has the advantages of a comparable database, robust discrimination, and relatively low cost. Our recent work shows that three-locus (*gyrB*-16S rRNA-*secA1*) MLSA for identification of clinical *Nocardia* species is superior to five-locus (*gyrB*-16S rRNA-*secA1*-*hsp65*-*rpoB*) MLSA which leads to misidentification for *N. abscessus* confirmed by digital DNA-DNA hybridization [17]. Therefore, the three-locus MLSA was carried out in this study to accurately identify clinical isolates from three tertiary care centers.

Although trimethoprim/sulfamethoxazole (SXT) has long been considered as the primary choice for therapy, it combines with other antibiotics that are always used for severe or systemic infections [12]. It is essential to carry out accurately antimicrobial susceptibility tests for clinical *Nocardia* isolates in China due to not only treatment for the individual patients but also providing guidance on empirical therapy in China.

The broth microdilution (BMD) method was recommended by the Clinical and Laboratory Standards Institute (CLSI) to determine antimicrobial susceptibility of *Nocardia* isolates [18], but few research groups carried out the susceptibility test with a relatively large number of isolates using this method in China (< 30 strains in the related references) [19–21]. Therefore, this study aimed to provide the susceptibility profile of 82 clinical *Nocardia* isolates using the BMD method.

## Results

### Species distribution and geographic characteristics

**Fig. 1:**
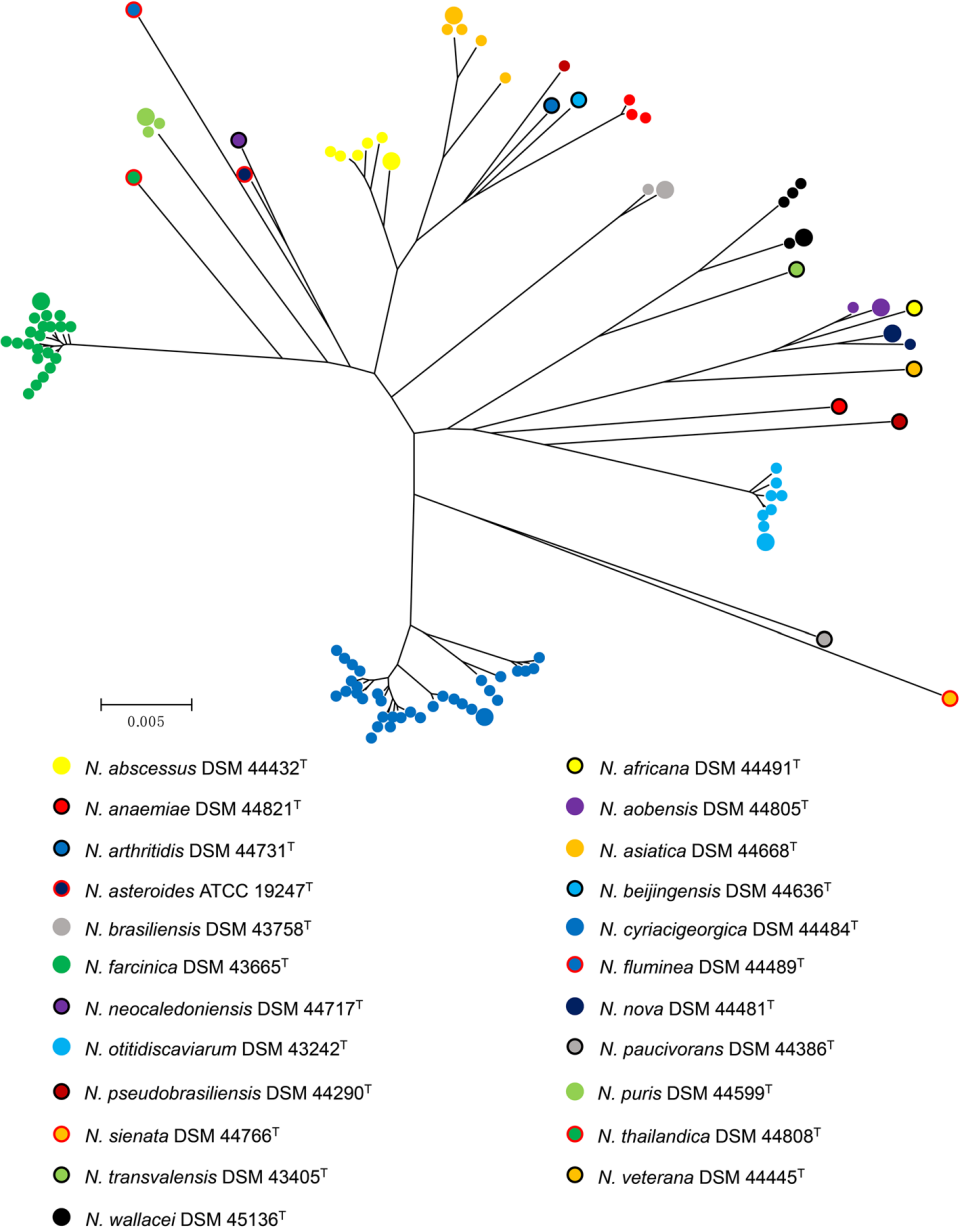
Phylogenetic neighbor-joining tree was based on the MLSA analysis from the 1902-bp concatenated *gyrB*-16S rRNA-*secA1* of the 23 *Nocardia* type strains and 82 *Nocardia* clinical strains. The isolates within each species cluster were assigned a color category according to their type strains. The big and small ball represented the type and clinical strains, respectively. The reliability of the topologies was assessed by the bootstrap method with 1000 replicates. ^T^, type strain

In the phylogenetic tree formed by three-locus MLSA (Fig. 1), these sequence clusters were considered to represent species clusters [14]. A phylogenetic tree was constructed from the 1902-bp concatenated *gyrB*-16S rRNA-*secA1* sequences of 23 *Nocardia* type strains and 82 clinical *Nocardia* isolates by the neighbor-joining method [22] and Kimura two-parameter distances [23].

Among the 82 isolates, 12 different species were represented. The two most common species were *N. cyriacigeorgica* (*n* = 33; 40.2%) and *N. farcinica* (*n* = 20; 24.4%). Other species included *N. otitidiscaviarum* (*n* = 7; 8.5%), *N. abscessus* (*n* = 5; 6.1%), *N. asiatica* (*n* = 4; 4.9%), *N. wallacei* (*n* = 4; 4.9%), *N. puris* (*n* = 2; 2.4%), *N. aobensis* (*n* = 1; 1.2%), *N. brasiliensis* (*n* = 1; 1.2%), *N. nova* (*n* = 1; 1.2%), novel species I (*n* = 3; 3.7%) and novel species II (*n* = 1; 1.2%) (Table 1). The Novel species I and II were confirmed by digital DNA-DNA hybridization in our previous work because identification by five-locus MLSA and 16S rRNA gene phylogenetic tree had different results for these strains [17]. The majority of the *Nocardia* isolates were from the respiratory tract (*n* = 74; 90.2%), followed by superficial abscess (*n* = 3; 3.7%), blood (*n* = 2; 2.4%), cerebrospinal fluid (CSF, *n* = 2; 2.4%), and joint fluid (*n* = 1; 1.2%). *N. cyriacigeorgica* (*n* = 32; 43.2%) was the most frequently isolated *Nocardia* spp. from the respiratory tract. The isolates from blood, CSF and joint fluid were all *N. farcinica* (*n* = 5; 100%).

**Table 1:** Antimicrobial susceptibility and MIC values (μg/ml) for clinical *Nocardia* isolates

Antimicrobial agents	Species (no. of isolates tested)												
	*N. cyriacigeorgica* (33)	*N. farcinica* (20)	*N. otitidiscaviarum* (7)	*N. abscessus* (5)	*N. asiatica* (4)	*N. wallacei* (4)	*N. puris* (2)	*N. aobensis* (1)	*N. brasiliensis* (1)	*N. nova* (1)	Novel species I (3)	Novel species II (1)	All *Nocardia* isolates (82)
Amoxicillin / clavulanic acid (S: ≤8/4 μg/ml; I: 16/8 μg/ml; R: ≥32/16 μg/ml) ^a^													
MIC range / value	4/2- > 64/32	4/2–16/8	32/16–64/32	≤2/1	64/32- > 64/32	2/1–8/4	16/8–32/16	4/2	4/2	64/32	≤2/1- > 64/32	4/2	≤2/1- > 64/32
MIC_50_ / MIC_90_	16/8 / 64/32	8/4 / 8/4	64/32 / 64/32	≤2/1 / ≤2/1	64/32 / > 64/32	4/2 / 8/4	–	–	–	–	–	–	16/8 / 64/32
S [n (%)]	2 (6.1)	18 (90.0)	0	5 (100)	0	4 (100)	0	1 (100)	1 (100)	0	1 (33.3)	1 (100)	33 (40.2)
I [n (%)]	18 (54.5)	2 (10.0)	0	0	0	0	1 (50.0)	0	0	0	0	0	21 (25.6)
R [n (%)]	13 (39.4)	0	7 (100)	0	4 (100)	0	1 (50.0)	0	0	1 (100)	2 (66.7)	0	28 (34.1)
Ceftriaxone (S: ≤8 μg/ml; I: 16–32 μg/ml; R: ≥64 μg/ml)													
MIC range / value	≤4- > 64	16- > 64	> 64	≤4	≤4	≤4–8	> 64	8	16	≤4	≤4	16	≤4- > 64
MIC_50_ / MIC_90_	8 / > 64	64 / > 64	> 64 / > 64	≤4 / ≤4	≤4 / ≤4	≤4 / 8	–	–	–	–	–	–	16 / > 64
S [n (%)]	17 (51.5)	0	0	5 (100)	4 (100)	4 (100)	0	1 (100)	0	1 (100)	3 (100)	0	35 (42.7)
I [n (%)]	8 (24.2)	5 (25.0)	0	0	0	0	0	0	1 (100)	0	0	1 (100)	15 (18.3)
R [n (%)]	8 (24.2)	15 (75.0)	7 (100)	0	0	0	2 (100)	0	0	0	0	0	32 (39.0)
Cefepime (S: ≤8 μg/ml; I: 16 μg/ml; R: ≥32 μg/ml)													
MIC range / value	4- > 32	16- > 32	> 32	≤1–8	≤1–8	≤1–32	> 32	2	32	4	≤1–2	16	≤1- > 32
MIC_50_ / MIC_90_	16 / > 32	> 32 / > 32	> 32 / > 32	4 / 8	4 / 8	4 / 32	–	–	–	–	–	–	32 / > 32
S [n (%)]	11 (33.3)	0	0	5 (100)	4 (100)	3 (75.0)	0	1 (100)	0	1 (100)	3 (100)	0	28 (34.1)
I [n (%)]	6 (18.2)	2 (10.0)	0	0	0	0	0	0	0	0	0	1 (100)	9 (11.0)
R [n (%)]	16 (48.5)	18 (90.0)	7 (100)	0	0	1 (25.0)	2 (100)	0	1 (100)	0	0	0	45 (54.9)
Imipenem (S: ≤4 μg/ml; I: 8 μg/ml; R: ≥16 μg/ml)													
MIC range / value	≤2–32	≤2–16	16- > − 64	≤2–8	≤2	≤2–32	4–8	≤2	64	≤2	≤2	> 64	≤2- > 64
MIC_50_ / MIC_90_	≤2 / 16	≤2 / 16	64 / > 64	4 / 8	≤2 / ≤2	≤2 / 32	–	–	–	–	–	–	≤2 / 32
S [n (%)]	29 (87.9)	15 (75.0)	0	3 (60.0)	4 (100)	3 (75.0)	1 (50.0)	1 (100)	0	1 (100)	3 (100)	0	60 (73.2)
I [n (%)]	0	0	0	2 (40.0)	0	0	1 (50.0)	0	0	0	0	0	3 (3.7)
R [n (%)]	4 (12.1)	5 (25.0)	7 (100)	0	0	1 (25.0)	0	0	1 (100)	0	0	1 (100)	19 (23.2)
Cefoxitin													
MIC range / value	16- > 128	64- > 128	> 128	≤4–8	≤4–32	64- > 128	64–128	64	128	64	≤4–16	32	≤4- > 128
MIC_50_ / MIC_90_	> 128 / > 128	128 / > 128	> 128 / > 128	8 / 8	8 / 32	128 / > 128	–	–	–	–	–	–	128 / > 128
Tobramycin (S: ≤4 μg/ml; I: 8 μg/ml; R: ≥16 μg/ml)													
MIC range/value	≤1–8	16- > 16	≤1–8	≤1	≤1–2	> 16	4–8	2	2	> 16	≤1–4	≤1	≤1- > 16
MIC_50_ / MIC_90_	≤1 / 4	> 16 / > 16	2 / 8	≤1 / ≤1	≤1 / 2	> 16 / > 16	–	–	–	–	–	–	2 / > 16
S [n (%)]	30 (90.9)	0	6 (85.7)	5 (100)	4 (100)	0	1 (50.0)	1 (100)	1 (100)	0	3 (100)	1 (100)	52 (63.4)
I [n (%)]	3 (9.1)	0	1 (14.3)	0	0	0	1 (50.0)	0	0	0	0	0	5 (6.1)
R [n (%)]	0	20 (100)	0	0	0	4 (100)	0	0	0	1 (100)	0	0	25 (30.5)
Amikacin (S: ≤8 μg/ml; R: ≥16 μg/ml)													
MIC range/value	≤1–4	≤1–8	≤1–4	≤1	≤1–2	16- > 64	≤1	≤1	2	≤1	≤1–2	≤1	≤1- > 64
MIC_50_ / MIC_90_	≤1 / 2	≤1 / 2	2 / 4	≤1 / ≤1	2 / 2	16 / > 64	–	–	–	–	–	–	≤1 / 2
S [n (%)]	33 (100)	20 (100)	7 (100)	5 (100)	4 (100)	0	2 (100)	1 (100)	1 (100)	1 (100)	3 (100)	1 (100)	78 (95.1)
R [n (%)]	0	0	0	0	0	4 (100)	0	0	0	0	0	0	4 (4.9)
Minocycline (S: ≤1 μg/ml; I: 2–4 μg/ml; R: ≥8 μg/ml)													
MIC range/value	≤1–4	≤1–4	≤1–2	≤1	≤1	≤1–4	≤1	≤1	≤1	2	≤1–2	≤1	≤1–4
MIC_50_ / MIC_90_	2 / 4	2 / 2	≤1 / 2	≤1 / ≤1	≤1 / ≤1	1 / 4	–	–	–	–	–	–	2 / 2
S [n (%)]	8 (24.2)	3 (15.0)	6 (85.7)	5 (100)	4 (100)	2 (50.0)	2 (100)	1 (100)	1 (100)	0	2 (66.7)	1 (100)	35 (42.7)
I [n (%)]	25 (75.8)	17 (85.0)	1 (14.3)	0	0	2 (50.0)	0	0	0	1 (100)	1 (33.3)	0	47 (57.3)
R [n (%)]	0	0	0	0	0	0	0	0	0	0	0	0	0
Doxycycline (S: ≤1 μg/ml; I: 2–4 μg/ml; R: ≥8 μg/ml)													
MIC range/value	0.25- > 16	0.25- > 16	0.25–4	≤0.12–1	0.24–4	0.25–8	0.5–1	4	2	8	0.5–4	≤0.12	≤0.12- > 16
MIC_50_ / MIC_90_	2 / 4	4 / 8	2 / 4	0.5 / 1	1 / 4	4 / 8	–	–	–	–	–	–	2 / 4
S [n (%)]	4 (12.1)	1 (5.0)	2 (28.6)	5 (100)	2 (50.0)	1 (25.0)	2 (100)	0	0	0	2 (66.7)	1 (100)	20 (24.4)
I [n (%)]	27 (81.8)	15 (75.0)	5 (71.4)	0	2 (50.0)	2 (50.0)	0	1 (100)	1 (100)	0	1 (33.3)	0	54 (65.9)
R [n (%)]	2 (6.1)	4 (20.0)	0	0	0	1 (25.0)	0	0	0	1 (100)	0	0	8 (9.8)
Tigecycline													
MIC range/value	0.06- > 4	2–4	0.25–2	1–2	0.5–2	0.5–4	1	1	1	2	1–2	0.5	0.06- > 4
MIC_50_ / MIC_90_	2 / 2	2 / 4	0.5 / 2	1 / 2	1 / 2	1 / 4	–	–	–	–	–	–	2 / 4
Ciprofloxacin (S: ≤1 μg/ml; I: 2 μg/ml; R: ≥4 μg/ml)													
MIC range/value	4- > 4	0.25- > 4	2- > 4	2- > 4	> 4	0.5- > 4	4- > 4	> 4	> 4	2	> 4	0.25	0.25- > 4
MIC_50_ / MIC_90_	> 4 / > 4	1 / > 4	> 4 / > 4	> 4 / > 4	> 4 / > 4	1 / > 4	–	–	–	–	–	–	> 4 / > 4
S [n (%)]	0	11 (55.0)	0	0	0	2 (50.0)	0	0	0	0	0	1 (100)	14 (17.1)
I [n (%)]	0	4 (20.0)	1 (14.3)	1 (20.0)	0	1 (25.0)	0	0	0	1 (100)	0	0	8 (9.8)
R [n (%)]	33 (100)	5 (25.0)	6 (85.7)	4 (80.0)	4 (100)	1 (25.0)	2 (100)	1 (100)	1 (100)	0	3 (100)	0	60 (73.2)
Moxifloxacin (S: ≤1 μg/ml; I: 2 μg/ml; R: ≥4 μg/ml)													
MIC range/value	1- > 8	≤0.25–4	1–4	1–8	> 8	0.5–2	2	2	1	2	> 8	≤0.25	≤0.25- > 8
^MIC^_50_^/ MIC^_90_	4 / 8	1 / 2	4 / 4	2 / 8	> 8 / > 8	2 / 2	–	–	–	–	–	–	2 / 8
S [n (%)]	2 (6.1)	15 (75.0)	1 (14.3)	1 (20.0)	0	1 (25.0)	0	0	1 (100)	0	0	1 (100)	22 (26.8)
I [n (%)]	13 (39.4)	3 (15.0)	2 (28.6)	2 (40.0)	0	3 (75.0)	2 (100)	1 (100)	0	1 (100)	0	0	27 (32.9)
R [n (%)]	18 (54.5)	2 (10.0)	4 (57.1)	2 (40.0)	4 (100)	0	0	0	0	0	3 (100)	0	33 (40.2)
Clarithromycin (S: ≤2 μg/ml; I: 4 μg/ml; R: ≥8 μg/ml)													
MIC range/value	4- > 16	> 16	> 16	2- > 16	4- > 16	2- > 16	> 16	0.25	> 16	0.12	8- > 16	16	0.12- > 16
^MIC^_50_^/ MIC^_90_	> 16 / > 16	> 16 / > 16	> 16/ > 16	> 16 / > 16	> 16 / > 16	8 / > 16	–	–	–	–	–	–	> 16 / > 16
S [n (%)]	1 (3.0)	0	0	1 (20.0)	0	1 (25.0)	0	1 (100)	0	1 (100)	0	0	5 (6.1)
I [n (%)]	0	0	0	0	1 (25.0)	0	0	0	0	0	0	0	1 (1.2)
R [n (%)]	32 (97.0)	20 (100)	7 (100)	4 (80.0)	3 (75.0)	3 (75.0)	2 (100)	0	1 (100)	0	3 (100)	1 (100)	76 (92.7)
Linezolid (S: ≤8 μg/ml)													
MIC range/value	≤1–4	≤1–4	1 ≤ −4	≤1–2	≤1–2	≤1–2	≤1–2	2	≤1	≤1	1–2	≤1	≤1–4
^MIC^_50_^/ MIC^_90_	2 / 2	2 / 4	≤1 / 4	≤1 / 2	≤1 / 2	≤1 / 2	–	–	–	–	–	–	2 / 2
S [n (%)]	33 (100)	20 (100)	7 (100)	5 (100)	4 (100)	4 (100)	2 (100)	1 (100)	1 (100)	1 (100)	3 (100)	1 (100)	82 (100)
Trimethoprim / sulfamethoxazole (S: ≤2/38 μg/ml; R: ≥4/76 μg/ml)													
MIC range/value	≤0.25/4.75–0.5/9.5	≤0.25/4.75–0.5/9.5	1/19–2/38	≤0.25/4.75	≤0.25/4.75–0.5/9.5	0.5/9.5–8/152	1/19–2/38	≤0.25/4.75	≤0.25/4.75	0.5/9.5	≤0.25/4.75–1/19	≤0.25/4.75	≤0.25/4.75–8/152
^MIC^_50_^/ MIC^_90_	≤0.25/4.75 / ≤0.25/4.75	≤0.25/4.75 / 0.5/9.5	2/38 / 2/38	≤0.25/4.75 / ≤0.25/4.75	≤0.25/4.75 / 0.5/9.5	1/19 / 8/152	–	–	–	–	–	–	≤0.25/4.75 / 2/38
S [n (%)]	33 (100)	20 (100)	7 (100)	5 (100)	4 (100)	3 (75.0)	2 (100)	1 (100)	1 (100)	1 (100)	3 (100)	1 (100)	81 (98.8)
R [n (%)]	0	0	0	0	0	1 (25.0)	0	0	0	0	0	0	1 (1.2)

^a^Broth microdilution breakpoints (μg/ml) of 13 antimicrobial agents for *Nocardia* according to the CLSI interpretive criteria [18]. MIC, minimum inhibitory concentration; MIC_50/90_, MIC for 50 and 90% of the isolates, respectively

**Fig. 2:**
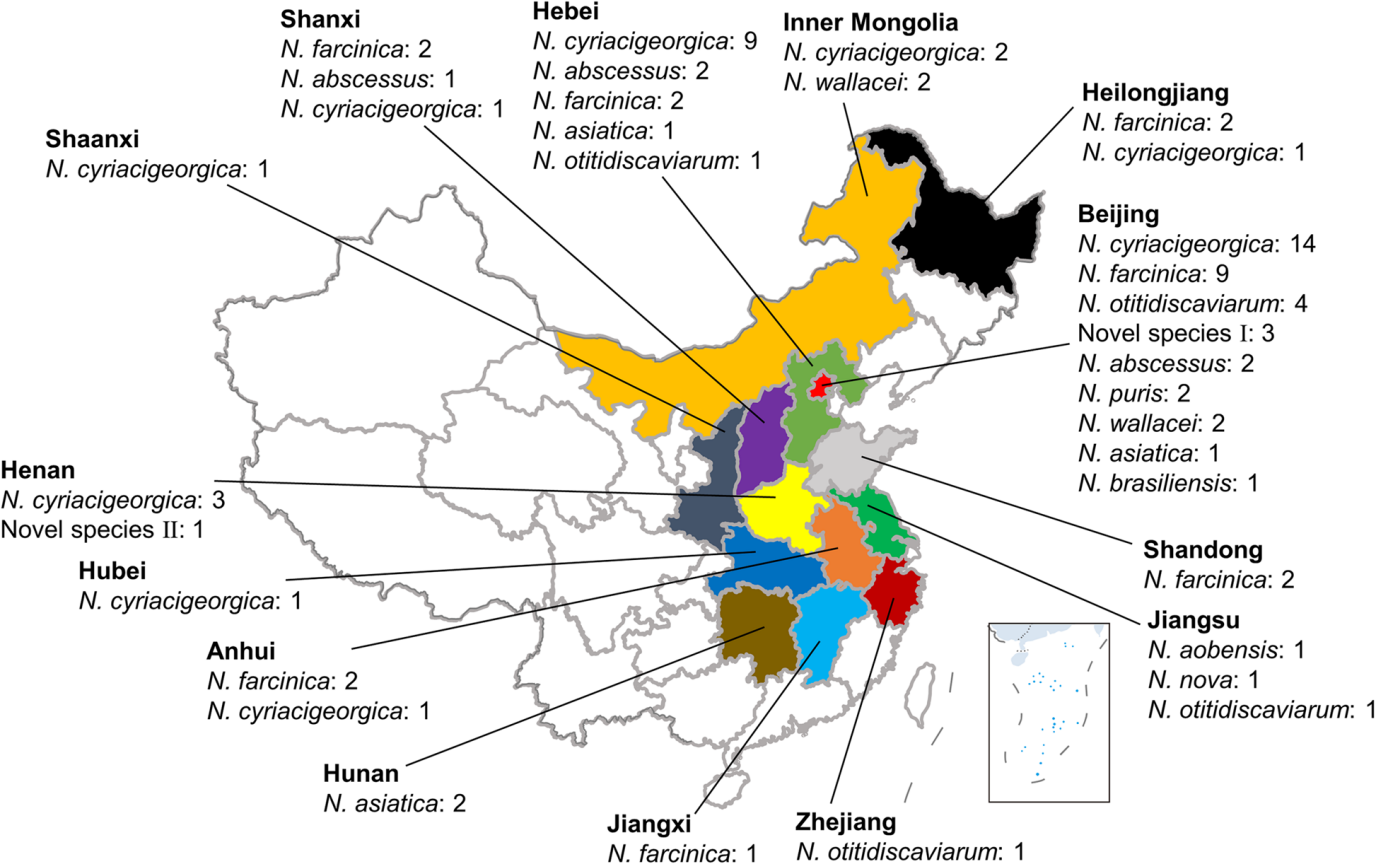
Geographic distribution of 82 clinical isolates of *Nocardia* in this study. The map of China was from the following website: http://bzdt.ch.mnr.gov.cn/, and the figure was finalized by adobe illustrator cs5

The geographic distribution of the clinical isolates is shown in Fig. 2. *N. cyriacigeorgica* and *N. farcinica* were the most widely distributed species in this study, being distributed in 64.3% (9/14) and 57.1% (8/14) of provinces, respectively. *N. otitidiscaviarum* isolates were distributed in eastern coastal regions (Beijing, Hebei, Jiangsu, and Zhejiang). *N. abscessus* isolates were distributed in three adjacent provinces of north China (Beijing, Hebei, and Shanxi).

### Demographic characteristics and infection types.

The demographic characteristics and infection types of 82 patients with nocardiosis are shown in Table 2. More than half of the patients age ranged from 50 to 69 years old. Of the 82 patients, pulmonary infection was the primary type of infection.

**Table 2:** Demographic characteristics and infection types of 82 patients with nocardiosis

Characteristic	n ^a^	%
Age (Mean ± S.D.)	56.3 ± 15.7	–
20–49	22	26.8
50–69	47	57.3
70–89	13	15.9
Sex		
Male	49	59.8
Female	33	40.2
Infection type		
Disseminated infection ^b^	5	6.1
Pulmonary infection	74	90.2
Superficial infection	3	3.7

*S.D* Standard deviation. -, not applicable

^a^Data are n (%) unless otherwise stated

^b^ The disseminated infection not involved the lung

### Antimicrobial susceptibility testing

There are no CLSI clinical breakpoints for cefoxitin and tigecycline, but the studied 82 *Nocardia* clinical strains showed an overall susceptibility rate of the other 13 antimicrobial drugs according to species that is summarized in Table 1. In addition, the MIC range or value, MIC_50_ and MIC_90_ values for the 82 isolates are also summarized in Table 1. The isolates demonstrated 98.8 and 100% susceptibility to SXT and linezolid, respectively. The susceptibility rates to amikacin, imipenem, tobramycin, ceftriaxone, minocycline, and amoxicillin/clavulanic acid were 95.1, 73.2, 63.4, 42.7, 42.7, and 40.2%, respectively, whilst the resistance rates to clarithromycin, ciprofloxacin, cefepime and moxifloxacin was 92.7, 73.2, 54.9, and 40.2%. Although a low resistance level to doxycycline (9.8%) was observed, a high percentage of isolates was in the intermediate category (65.9%). The MIC_50_ and MIC_90_ for cefoxitin (128 and > 128 μg/ml) of all *Nocardia* spp. were high, but for tigecycline (2 and 4 μg/ml) were low, which is similar to the report by Tan et al. [24]

As previously described [12, 25], we noted a strong correlation between the drug susceptibility pattern types and *Nocardia* species. The type I, III, IV, V, and VI were displayed by the *N. abscessus*, *N. nova*, *N. wallacei*, *N. farcinica*, and *N. cyriacigeorgica*, respectively. In addition, the species that were not defined drug susceptibility pattern types also had unique drug susceptibility profiles. The susceptibility profiles were varied by species of *Nocardia* which are shown in Table S1. The susceptibility rates to amoxicillin/clavulanic acid and moxifloxacin were high for *N. farcinica* (90 and 75%), but they were low for *N. cyriacigeorgica* (6.1 and 6.1%), *N. otitidiscaviarum* (0 and 14.3%), and *N. asiatica* (0 and 0), respectively. *N. wallacei* was the only species that was resistant to amikacin. Imipenem was effective against most *Nocardia* spp., but it was ineffective against *N. otitidiscaviarum* isolates.

Table S1 also shows that the drug susceptibility patterns reported by Wallace et al. [26] and Schlaberg et al. [25] The similarities and slight differences are all displayed.

## Discussion

Due to the large number of *Nocardia* species, biochemical methods are insufficient to identify the clinically relevant species [4]. MALDI-TOF MS can rapidly identify the frequently encountered *Nocardia* species, but it is limited by the database and distinguishing closely related species [10]. The 16S rRNA gene sequencing is robust for accurate identification of *Nocardia* species, but it is also limited by discriminating closely related species due to high conservation [12]. The current study accurately identified the species by MLSA and determined the antimicrobial susceptibility by BMD method of clinically isolated *Nocardia* from three tertiary hospitals in China. The MLSA which is promising as the primary method in identification of prokaryotic species has powerful interspecies and intraspecies discrimination [14, 15, 27]. In our previous work, a fact has been proved by digital DNA-DNA hybridization analysis that three-locus MLSA is superior to five-locus MLSA for *Nocardia* species identification [17]. Therefore, three-locus MLSA was carried out in this study.

The geographical distribution of the *Nocardia* species has unique characteristics around the world. Figure 1 shows that *N. cyriacigeorgica* (40.2%) was the most encountered species in this study that is similar to the reports from Iran, Spain, and the USA [9, 28, 29]. The prevalent species are slightly different in different regions [28, 30, 31]. Even within China, the prevalent species also have regional characteristics. *N. otitidiscaviarum* appears to be more prevalent in the eastern and southern coastal areas of China, and *N. abscessus* prefers to be distributed in the neighboring northern provinces of China, including Shanxi, Hebei, Beijing, and Shandong [20, 32]. *N. aobensis* and *N. nova* which are close species on 16S rRNA and/or *secA1* genes and even classified into *N. nova* complex by Conville et al. [4], are distributed in Jiangsu in this study, while *N. nova* is distributed in Shandong in the study by Huang et al. [32] Actually, Jiangsu and Shandong are adjacent to each other. *N. asiatica* is mainly distributed in Hunan in this study, while it is distributed in Chongqing and Guangxi, which are adjacent to Hunan in the study by Huang et al. [32]

Based on the current study and the related references mentioned above [20, 32], an interesting phenomenon was found: Some species prefer to be distributed according to the climate type, while others prefer to be distributed along the coast in China. Shanxi, Hebei, Beijing, and Shandong all belong to the monsoon climate of medium latitudes, and *N. abscessus* is prevalent. Hunan, Chongqing, and Guangxi all belong to the subtropical monsoon climate, and *N. asiatica* is prevalent. *N. otitidiscaviarum* and *N. nova* complex tend to be distributed in coastal provinces (Beijing, Hebei, Shandong, Jiangsu, Zhejiang, and Guangxi). This phenomenon suggests that the distribution of *Nocardia* is affected by the climate type and the sea.

With the increase of sample size, the incidence of nocardiosis in men increased significantly, which is different from our previous conclusion that there is no difference between men and women [19], but the new finding is similar to the report by Martínez-Barricarte, who summarized the gender distribution of patients with isolated nocardiosis worldwide [33]. According to Hernandez Hernandez et al., the female hormone estradiol shows inhibitory effect on *Nocardia brasiliensis* [34], which suggests the gender difference may be caused by estradiol. The proportion of nocardiosis for age ranging from 50 to 69 years was more than half in this study (58.5%), which is similar to the report by Huang et al. (54.7%) [32], but it is different from the data reported by Martínez-Barricarte that age ranging from 31 to 40 and from 51 to 60 years are the maximum proportion [33]. It suggests that nocardiosis in China has unique characteristics in terms of age. Nocardiosis most often shows up as a pulmonary infection [1, 12], confirmed by this study (90.2%).

There are few large-scale studies [20, 21] about the antimicrobial susceptibility pattern of various *Nocardia* species based on BMD method that is recommended by the CLSI [18] in China. This study provides a relatively large collection of clinical *Nocardia* isolates to explore the correlation between antibiotics and species and reach a guideline for the nocardiosis treatment in China. SXT is the drug of the first choice for the treatment of nocardiosis, but some studies report a high level of resistance to the drug [35]. It is urgent to survey the SXT susceptibility in China. Overall, 98.8% of isolates were susceptible to SXT, and only one isolate of *N. wallacei* was resistant to the drug (Table 1). Similar results are reported by Lu et al. and other researchers [20, 36–39]. Therefore, the present study indicates that SXT remains high-level of activity against *Nocardia* in vivo and can still be considered as the first-line therapeutic drug of choice for nocardiosis in China. The discrepancy may be caused by geographic differences, and/or SXT exposure before testing [36]. According to the reports [28, 31], the isolation rates of *N. nova* complex and *N. farcinica* which have high resistance rates to SXT were more than 10% in Spain and the USA. However, the former is rarely isolated in China [20, 32], and the latter may be divided into different types due to the different geographic areas.

Linezolid, that shows 100% activity against clinical *Nocardia* isolates in several large-scale studies [24, 25, 35, 38, 40], has become popular in treating nocardiosis recently, and the present study showed the same results. Amikacin is also an effective drug for all *Nocardia* isolates except for *N. transvalensis* complex, which is intrinsic resistant to the drug [37, 38, 40–42]. However, *N. transvalensis* complex is not prevalent in China [20, 32]. In the study, *N. wallacei*, which belongs to *N. transvalensis* complex, is the only resistant species to amikacin. The treatment of nocardiosis commonly requires a combination of antibiotics. Imipenem is usually used in combination with SXT for adequate therapy of invasive *Nocardia* infections [43]. Imipenem showed good activity (susceptibility rate: 73.2%) in the current study which supports it to participate in the combination regimen in empirical therapy. Amoxicillin/clavulanic acid, minocycline, or doxycycline can be also selected in a combination therapy, because the frequently encountered *Nocardia* species exhibit low-level resistance to the three antibiotics in China.

However, the remaining antimicrobials showed low activity against *Nocardia* isolates, and the susceptibility had species-specific. Compared with the studies by Wallace et al. [26] and Schlaberg et al. [25], a similar correlation between the antimicrobials and *Nocardia* species is observed, but there are some differences as well (Table S1). In particular, Wallace et al. reported that *N. abscessus* was resistant to imipenem, but the resistance rates were 40% in this study and 69% in the study by Schlaberg et al. Unlike Wallace et al. and Schlaberg et al., who indicated that *N. wallacei* was susceptible to ciprofloxacin, the current study indicated 50% susceptibility rate to the drug. For *N. farcinica*, Wallace et al. and this study reported that it was susceptible to imipenem, while Schlaberg et al. reported that the susceptibility rate was only 33%. Besides, ciprofloxacin was less active to *N. farcinica* in the study by Schlaberg et al. and the current study, but was active in the study by Wallace et al. However, moxifloxacin, which is a higher generation quinolone antibiotic, was much more active than ciprofloxacin against *N. farcinica* in the study by Schlaberg et al. (susceptibility rate: 79%) and the current study (75%). It may be caused by the longer exposure time of ciprofloxacin compared to moxifloxacin [44]. For *N. cyriacigeorgica*, this study indicated that it was susceptible to imipenem (87.9%), which is similar to the study by Wallace et al., but Schlaberg et al. reported the susceptibility rate was only 43%. For *N. otitidiscaviarum*, ciprofloxacin was much active in the study by Wallace et al., but it was almost inactive in the study by Schlaberg et al. and the present study. The small differences mentioned above need to be further confirmed because the reproducibility of the BMD method for susceptibility testing of *Nocardia* species is not always very stable reported by Conville et al. [45]

For empirical treatment of nocardiosis, clarithromycin should be avoided due to the high resistance rate in this study. Ciprofloxacin is much less active to *N. cyriacigeorgica*, and ceftriaxone, cefepime, and tobramycin are much less active to *N. farcinica*. Therefore, these antibiotics also should avoid being used unless the species has been identified and/or the susceptibility test has been done, as *N. cyriacigeorgica* and *N. farcinica* are the most prevalent strains in China.

In addition to the major epidemic strains in China, the isolation rate of *N. otitidiscaviarum* in eastern and southern coastal areas is relatively high. The use of β-lactam antibiotics should be paid attention to because of its high resistance rate to this kind of antibiotics [20, 32].

## Conclusion

In summary, *N. cyriacigeorgica* and *N. farcinica*, which are widely distributed in China, were the most frequently isolated species, as well as they were the most common species causing pulmonary infection in this study. The clinically isolated *Nocardia* spp. had diverse antimicrobial susceptibility patterns, which were similar to the reports by other groups elsewhere, but some differences were also observed. It indicates the specific characteristics and provides the basis of empirical therapy in China. According to the current study, SXT can still be the first choice due to the low resistance rate. Linezolid can be chosen when a patient is allergic to SXT, and amikacin and imipenem can be the choice in a combination regimen.

## Methods

### Strains

A total of 82 non-repetitive clinical isolates of *Nocardia* from three tertiary hospitals in Beijing were studied between 2010 and 2020; seventy were from Beijing Chao-Yang Hospital, of which 26 isolates have been tested for antimicrobial susceptibility with self-made drug susceptibility plates and published in our previous work [19], seven from Beijing Ditan Hospital, and five from Aerospace Center Hospital. The three hospitals have 1880, 1158, and 1050 beds, respectively. Observation of gram-positive beaded branching filaments on a direct gram-stained smear and positive Kinyoun acid-fast stain under the microscope, as well as white to orange colonies on culture plates, indicated that the isolates were presumptive *Nocardia* [46]. The type strains in this study were selected as previously described [17]. In short, 23 type strains were used to construct phylogenetic relationships based on three-locus MLSA (*gyrB*-16S rRNA-*secA1*), and their GenBank accession numbers were shown in Table S2. Most of the type strains were selected based on their similarity to 16S rRNA gene sequences of the clinical isolates.

### DNA extraction, PCR, and sequencing

The DNA was extracted by boiling method, which was the same as our previous work [17]. The primers of 16S rRNA gene, and the primers of *gyrB* gene and *secA1* gene referred to the work published by Carrasco et al. [13] and McTaggart et al. [16], respectively. The forward and reverse primers were listed in Table S3. The PCR experiments were carried out as previously described [13, 16]. An ABI 3730XL DNA sequencer (Applied Biosystems) was used to sequence the PCR products, and the SeqMan program in Lasergene 7.1 (DNASTAR, Inc., Madison, WI) was used to assemble the sequences.

### Construction of phylogenetic tree

The gene sequences of *gyrB*, 16S rRNA, and *secA1* was aligned and trimmed by Mega (version 6.0) software [47] to generate the fragments of 482 bp, 1026 bp, and 394 bp, respectively. The concatenation of *gyrB*-16S rRNA-*secA1* (1902 bp sequence) was used to constructed phylogenetic tree by Mega software. The tree was computed by the neighbor-joining method [22] and Kimura two-parameter distances [23]. The bootstrap method with 1000 replicates was used to ensure the reliability of the topologies.

### Antimicrobial susceptibility testing by broth microdilution method

Antimicrobial susceptibility testing of all isolates to 15 antimicrobial agents [amikacin (1–64 μg/ml), amoxicillin/clavulanic acid (2/1–64/32 μg/ml), cefepime (1–32 μg/ml), cefoxitin (4–128 μg/ml), ceftriaxone (4–64 μg/ml), ciprofloxacin (0.12–4 μg/ml), clarithromycin (0.06–16 μg/ml), doxycycline (0.12–16 μg/ml), imipenem (2–64 μg/ml), linezolid (1–32 μg/ml), minocycline (1–8 μg/ml), moxifloxacin (0.25–8 μg/ml), tigecycline (0.015–4 μg/ml), tobramycin (1–16 μg/ml), and trimethoprim/sulfamethoxazole (SXT) (0.25/4.75–8/152 μg/ml)] was determined by BMD using the commercial Sensititre™ RAPMYCOI (Thermo Scientific, the United States). Briefly, 50 μl of an organism suspension with a turbidity equivalent to ~ 0.5 McFarland standard was transferred to 10 ml of Mueller Hinton II Broth (Becton, Dickinson and Company, the United States), and then 100 μl was inoculated into the microdilution wells to give a final concentration of ~ 5 × 10^5^ CFU/ml [18]. The microtitre plates were incubated aerobically at 35 °C and were read after 3 days (or after 5 days if growth was insufficient after 3 days). Growth was examined daily by visual inspection. The minimum inhibitory concentration (MIC) was defined as the lowest concentration of drug that inhibited visible growth, except for SXT where the MIC was the 80% inhibition endpoint of growth compared with the control. Breakpoints for susceptibility and resistance were as defined by the CLSI (Table 1) [18]. *Nocardia asteroides* ATCC 19247, *Staphylococcus aureus* ATCC 29213 and *Escherichia coli* ATCC 35218 (for AMC only) were used as quality control strains.

## Supplementary Information

**Additional file 1: Table S1.** Comparison of drug susceptibility patterns with clinical *Nocardia* species. **Table S2.** GenBank accession numbers of gene sequences for *Nocardia* type strains in the study. **Table S3.** The primer sequences used in this study.

## Data Availability

The datasets generated and analysed during the current study are available in the Genome Sequence Archive (Genomics, Proteomics & Bioinformatics 2021) in National Genomics Data Center (Nucleic Acids Res 2021), China National Center for Bioinformation / Beijing Institute of Genomics, Chinese Academy of Sciences (GSA: CRA005268) that are publicly accessible at: https://bigd.big.ac.cn/gsa/browse/CRA005268.
