# Supplementary material for: MLSA phylogeny and antimicrobial susceptibility of clinical Nocardia isolates: a multicenter retrospective study in China
Source: BMC Microbiol. 2021 Dec 13;21:342. doi: 10.1186/s12866-021-02412-x (PMC8667443; doi:10.1186/s12866-021-02412-x)
Supplement: Supplementary file 1 — Additional file 1: Table S1. Comparison of drug susceptibility patterns with clinical Nocardia species. Table S2. GenBank accession numbers of gene sequences for Nocardia type strains in the study. Table S3. The primer sequences used in this study. [file 12866_2021_2412_MOESM1_ESM.docx]

**MLSA phylogeny and antimicrobial susceptibility of clinical *Nocardia* isolates: a multicenter retrospective study in China**

Ming Wei^1^, Xinmin Xu^2^, Jingxian Yang^3^, Peng Wang^1^, Yongzhe Liu^1^, Shuai Wang^1^, Chunxia Yang^1^, Li Gu^1,*^

^1^*Department of Infectious Diseases and Clinical Microbiology, Beijing Chao-Yang Hospital, Capital Medical University, Beijing, People's Republic of China*;

^2^*Department of Clinical Laboratory, Beijing Ditan Hospital, Capital Medical University, Beijing, People's Republic of China*;

^3^*Department of Clinical Laboratory, Aerospace Center Hospital, Beijing, People's Republic of China.*

**^*^Corresponding author:** Li Gu, M.D. Department of Infectious Diseases and Clinical Microbiology, Beijing Chao-Yang Hospital, 8 Gongren Tiyuchang Nanlu, Chaoyang District, Beijing, 100020, People’s Republic of China

E-mail: guli2013227@foxmail.com

Phone/Fax: +86 10 8523 1130

**Table S1** Comparison of drug susceptibility patterns with clinical *Nocardia* species

| *Nocardia* Species | No. of isolates in this study | Drug susceptibility pattern types | Drug susceptibility pattern characteristics (only the drugs in this study are included) | | | | | | | | |
| --- | --- | --- | --- | --- | --- | --- | --- | --- | --- | --- | --- |
|  |  |  | Susceptible ^a^ | | |  | Resistant ^a^ | |  | Non-susceptible ^a^ | |
|  |  |  | Wallace et al. ^[1]^ | Schlaberg et al. ^[2]^ | This study |  | Wallace et al. ^[1]^ | This study |  | Schlaberg et al. ^[2]^ | This study |
| *N. abscessus* | 5 | Ⅰ | AMC; CRO; AMK; LZD | AMC; CRO; TOB; AMK; MIN; LZD; SXT | AMC; CRO; FEP; TOB; AMK; MIN; DOX; LZD; SXT |  | CIP; CLA; IPM | CIP; CLA |  | CIP; MXF; CLA | CIP; MXF; CLA |
| *N. nova* | 1 | Ⅲ | CRO; IPM; AMK; CLA; LZD | IPM; AMK; CLA; LZD; SXT | CRO; FEP; IPM; AMK; CLA; LZD; SXT |  | AMC | AMC; TOB; DOX |  | AMC; TOB; MIN; CIP; MXF | AMC; TOB; MIN; DOX; CIP; MXF |
| *N. wallacei* | 4 | Ⅳ | CRO; IPM; CIP; LZD; | CIP; MXF; LZD; SXT | AMC; CRO; FEP; IPM; LZD; SXT |  | TOB; AMK; CLA | TOB; AMK; CLA |  | IPM; TOB; AMK; MIN; CLA | TOB; AMK; DOX; MXF; CLA |
| *N. farcinica* | 20 | Ⅴ | IPM; AMK; CIP; LZD | AMC; AMK; MXF; LZD; SXT | AMC; IPM; AMK; MXF; LZD; SXT |  | CRO; FEP; TOB; CLA | CRO; FEP; TOB; CLA |  | CRO; TOB; MIN; CLA | CRO; FEP; TOB; MIN; DOX; CLA |
| *N. cyriacigeorgica* | 33 | Ⅵ | CRO; IPM; AMK; LZD | CRO; TOB; AMK; LZD; SXT | IPM; TOB; AMK; LZD; SXT |  | AMC; CIP; CLA | CIP; CLA |  | AMC; MIN; CIP; MXF CLA | AMC; MIN; DOX; CIP; MXF; CLA |
| *N. otitidiscaviarum* | 7 | NA | AMK; CIP; SXT | AMK; LZD; SXT | TOB; AMK; MIN; LZD; SXT |  | all β-lactam antibiotics | AMC; CRO; FEP; IPM; CIP |  | AMC; CRO; IPM; CIP; CLA | AMC; CRO; FEP; IPM; DOX; CIP; MXF; CLA |
| *N. asiatica* | 4 | NA | NA | NA | CRO; FEP; IPM; TOB; AMK; MIN; LZD; SXT |  | NA | AMC; CIP; MXF; CLA |  | NA | AMC; CIP; MXF; CLA |
| *N. puris* | 2 | NA | NA | TOB; AMK; MIN; LZD; SXT | AMK; MIN; DOX; LZD; SXT |  | NA | CRO; FEP; CIP; CLA |  | AMC; CRO; CIP; MXF; CLA | AMC; CRO; FEP; CIP; MXF; CLA |
| *N. aobensis* | 1 | NA | NA | AMK; CLA LZD; SXT | AMC; CRO; FEP; IPM; TOB; AMK; MIN; CLA; LZD; SXT |  | NA | CIP |  | AMC; CIP; MIN; TOB; MXF | DOX; CIP; MXF |
| *N. brasiliensis* | 1 | NA | AMC; MIN; SXT | AMC; TOB; AMK; MXF; LZD; SXT | AMC; TOB; AMK; MIN; MXF; LZD; SXT |  | CIP; CLA | FEP; IPM; CIP; CLA |  | IPM; MIN; CIP; CLA | CRO; FEP; IPM; DOX; CIP; CLA |
| Novel species Ⅰ ^b^ | 3 | NA | NA | CRO; IPM; TOB; AMK; MIN; LZD; SXT | CRO; FEP; IPM; TOB; AMK; LZD; SXT |  | NA | CIP; MXF; CLA |  | AMC; CIP; MXF | CIP; MXF; CLA |
| Novel species Ⅱ | 1 | NA | NA | NA | AMC; TOB; AMK; MIN; DOX; CIP; MXF; LZD; SXT |  | NA | IPM; CLA |  | NA | CRO; FEP; IPM; CLA |

^a^ The proportion of isolates ≥ 70% was defined as susceptible, resistant, and non-susceptible in this study; ^b^ Novel species Ⅰ in this study compared with *N. beijingensis* complex in the study by Schlaberg et al. Amoxicillin/clavulanic acid, AMC; Ceftriaxone, CRO; Cefepime, FEP; Imipenem, IPM; Tobramycin, TOB; Amikacin, AMK; Minocycline, MIN; Doxycycline, DOX; Ciprofloxacin, CIP; Moxifloxacin, MXF; Clarithromycin, CLA; Linezolid, LZD; Trimethoprim/sulfamethoxazole, SXT.

**Table S2** GenBank accession numbers of gene sequences for *Nocardia* type strains in the study

| Type strain | GenBank accession number | | |
| --- | --- | --- | --- |
|  | 16S rRNA | *gyrB* | *secA1* |
| *N. abscessus* DSM 44432^T^ | GQ376194.1 | JN041252.1 | JN041963.1 |
| *N. africana* DSM 44491^T^ | NR_041872.1 | JN042222.2 | JN042079.1 |
| *N. anaemiae* DSM 44821^T^ | NR_118197.1 | JN041295.1 | EU178743.1 |
| *N. aobensis* DSM 44805^T^ | NR_118198.1 | JN041378.1 | EU178744.1 |
| *N. arthritidis* DSM 44731^T^ | NR_115824.1 | JN041235.1 | JN041946.1 |
| *N. asiatica* DSM 44668^T^ | NR_028644.1 | JN041250.1 | DQ085139.1 |
| *N. asteroides* ATCC 19247^T^ | NR_115826.1 | JN041222.1 | DQ360267.1 |
| *N. beijingensis* DSM 44636^T^ | NR_118618.1 | JN041231.1 | JN041942.1 |
| *N. brasiliensis* DSM 43758^T^ | NR_119106.1 | JN041298.1 | JN042009.1 |
| *N. cyriacigeorgica* DSM 44484^T^ | NR_041857.1 | JN041323.1 | DQ360272.1 |
| *N. farcinica* DSM 43665^T^ | NR_114643.1 | KR068638.1 | KR068815.1 |
| *N. fluminea* DSM 44489^T^ | NR_114644.1 | JN041215.1 | JN041926.1 |
| *N. neocaledoniensis* DSM 44717^T^ | NR_118204.1 | JN041221.1 | JN041932.1 |
| *N. nova* DSM 44481^T^ | NR_041858.1 | JN041413.1 | JN042124.1 |
| *N. otitidiscaviarum* DSM 43242^T^ | NR_041874.1 | JN041275.1 | JN041986.1 |
| *N. paucivorans* DSM 44386^T^ | NR_041863.1 | JN041350.1 | JN042061.1 |
| *N. pseudobrasiliensis* DSM 44290^T^ | NR_041864.1 | JN041284.1 | JN041995.1 |
| *N. puris* DSM 44599 ^T^ | NR_118207.1 | NZ_QNRE01000004.1 | EU178750.1 |
| *N. sienata* DSM 44766^T^ | NR_118209.1 | JN041357.1 | JN042068.1 |
| *N. thailandica* DSM 44808^T^ | NR_118213.1 | JN041212.1 | EU178752.1 |
| *N. transvalensis* DSM 43405^T^ | NR_041867.1 | JN041286.1 | DQ360287.1 |
| *N. veterana* DSM 44445^T^ | NR_115842.1 | NZ_JAAXPE010000005.1 | DQ360288.1 |
| *N. wallacei* DSM 45136^T^ | NR_044401.1 | JN041290.1 | JN042001.1 |

Abbreviations: DSM, German Collection of Microorganisms and Cell Cultures; ATCC, American type culture collection; ^T^, Type strain.

**Table S3** The primer sequences used in this study

| Gene | Primer sequences | |
| --- | --- | --- |
|  | Forward primer | Reverse primer |
| 16S rRNA | 5’-GCTTAACACATGCAAGTCG-3’ | 5’-GAATTCCAGTCTCCCCTG-3’ |
| *gyrB* | 5'-CTTCGCCAACACCATCAACAC-3' | 5'-TGATGATCGACTGGACCTCG-3' |
| *secA1* | 5'-GCGACGCCGAGTGGATGG-3' | 5'-TTGGCCTTGATGGCGTTGTTC-3' |

**References**

[1] Wallace R J, Jr., Steele L C, Sumter G, and Smith J M. Antimicrobial susceptibility patterns of *Nocardia* asteroides. Antimicrob Agents Chemother. 1988, 32(12): 1776-1779.

[2] Schlaberg R, Fisher M A, and Hanson K E. Susceptibility profiles of *Nocardia* isolates based on current taxonomy. Antimicrob Agents Chemother. 2014, 58(2): 795-800.
